# Deep Learning-Based Speech Enhancement With a Loss Trading Off the Speech Distortion and the Noise Residue for Cochlear Implants

**DOI:** 10.3389/fmed.2021.740123

**Published:** 2021-11-08

**Authors:** Yuyong Kang, Nengheng Zheng, Qinglin Meng

**Affiliations:** ^1^Guangdong Key Laboratory of Intelligent Information Processing, College of Electronics and Information Engineering, Shenzhen University, Shenzhen, China; ^2^Pengcheng Laboratory, Shenzhen, China; ^3^Acoustics Laboratory, School of Physics and Optoelectronics, South China University of Technology, Guangzhou, China

**Keywords:** cochlear implant, speech enhancement, perceptual property, deep learning, loss function

## Abstract

The cochlea plays a key role in the transmission from acoustic vibration to neural stimulation upon which the brain perceives the sound. A cochlear implant (CI) is an auditory prosthesis to replace the damaged cochlear hair cells to achieve acoustic-to-neural conversion. However, the CI is a very coarse bionic imitation of the normal cochlea. The highly resolved time-frequency-intensity information transmitted by the normal cochlea, which is vital to high-quality auditory perception such as speech perception in challenging environments, cannot be guaranteed by CIs. Although CI recipients with state-of-the-art commercial CI devices achieve good speech perception in quiet backgrounds, they usually suffer from poor speech perception in noisy environments. Therefore, noise suppression or speech enhancement (SE) is one of the most important technologies for CI. In this study, we introduce recent progress in deep learning (DL), mostly neural networks (NN)-based SE front ends to CI, and discuss how the hearing properties of the CI recipients could be utilized to optimize the DL-based SE. In particular, different loss functions are introduced to supervise the NN training, and a set of objective and subjective experiments is presented. Results verify that the CI recipients are more sensitive to the residual noise than the SE-induced speech distortion, which has been common knowledge in CI research. Furthermore, speech reception threshold (SRT) in noise tests demonstrates that the intelligibility of the denoised speech can be significantly improved when the NN is trained with a loss function bias to more noise suppression than that with equal attention on noise residue and speech distortion.

## Introduction

A cochlear implant (CI) is an auditory prosthesis playing an essential role in restoring hearing ability for patients with severe-to-profound sensorineural hearing impairment (1, 2). CI recipients can achieve good speech understanding ability in quiet environments. However, their hearing ability degrades dramatically in noisy backgrounds (3, 4). The main reason is that the signal processing in CIs is a very coarse imitation of the sound coding in a healthy cochlea (5). The inner hair cells (around 3,500), in charge of transforming sound vibrations in the cochlea into electrical signals, are replaced by only 12–26 implanted intracochlear electrodes. Signal processing strategies in CIs can only transmit coarsely frequency-resolved temporal envelopes of the speech to stimulate the auditory nerves. Therefore, the information conveyed by the spectro-temporal fine structures, which are very important for speech understanding in noise, are not effectively represented in CI (6, 7). Therefore, speech enhancement (SE) algorithms have been developed to improve speech intelligibility in noisy environments for CI recipients (8–11). Unfortunately, this is still a pending problem.

Even for modern CIs with multiple microphones, single-channel SE algorithms are mostly implemented after a directional processing stage. Typical single-channel SE algorithms for CIs include spectral subtraction (SS), subspace projection (SP), Wiener filtering (WF), time-frequency (T-F) masking, etc. Yang et al. (12) implemented an SS-based SE as a front end to CI, which improved the speech understanding ability of CI recipients significantly in speech-shape noise (SSN) but not significantly in babble noise (Babble). Loizou et al. (13) proposed an SP-based SE where CI recipients received intelligibility improvement in stationary noise. Guevara et al. (14) proposed a multiband single-channel WF SE for CI recipients, where subjects achieved significant intelligibility gain in SSN but slightly improved in cocktail party noise. Koning et al. (15) investigated two T-F masking-based SEs: the ideal binary masking (IBM) and the ideal ratio masking (IRM), on their effectiveness in SE for CIs. Vocoder simulated tests showed that both maskings worked well given known *a priori* signal-to-noise ratio (SNR), but the performance cannot be guaranteed in real conditions due to the unavoidable SNR estimation error. Most of these traditional SE methods rely on an estimate of the noise (or SNR) and a prerequisite on noise stationarity. Therefore, their performances in nonstationary noise are usually not as convincing as in stationary noise.

Data-driven models, particularly the deep-learning (DL) ones, have been applied for SE with promising results, especially in nonstationary noisy environments where most conventional SEs fail. A well-known example is the spectral mapping-based SE, which uses the clean speech spectra as the training targets such that a noisy-to-clean spectral mapping network can be obtained (16). Another example is the masking-based SE, which is similar to the traditional IBM/IRM ones except that a network is trained to estimate the masking gain from the noisy input such that no explicit noise/SNR estimation is required (17). Model-based mapping or masking methods have also been adopted for SE in CI. In SE for CI, enhancement processing can be done on either the acoustic or electric signals. For the acoustic SE, Lai et al. (18, 19) proposed deep neural network (DNN)-based spectral mapping as an SE front end to CI processor. Both objective and subjective evaluations showed superior performance over traditional SEs. Goehring et al. (20) implemented the recurrent neural networks (RNN)-based T-F masking method to enhance the acoustic signal. Results indicated that both objective and subjective evaluations achieved significant improvement. For electric SE, Hu et al. (21) used a Gaussian mixture model (GMM) as the binary classifier to estimate the IBM gains for each electrode channel. Results demonstrated that CI subjects obtained significant improvement on speech understanding in Babble, Train, and Hall noises. Mamun etal. (22) proposed a convolutional neural network (CNN)-based IRM gain estimator to enhance the temporal envelopes (TEs) of each channel. Objective evaluations showed a significant improvement in speech intelligibility in noisy ambiance. Bolner et al. (23) and Goehring et al. (24) used DNN to estimate the electrode-dependent channel gains with which noise components in the TEs can be suppressed. Results showed that DNN-based IRM performed better than WF in both vocoder-simulated and CI-subjective tests. Zheng et al. (25) presented a DL-based CI strategy. Instead of serving as a front end or a built-in module in CI strategy, the NN was built and trained to simulate a specific strategy of a clinical CI device. The NN output was compatible with the clinical device, and the noise robustness of the NN was obtained through data-driven network optimization.

Most of the abovementioned DL-based SEs focus on minimizing the overall difference between the target speech and its denoised estimate, and, usually, the mean-square-error (MSE)-based loss functions are adopted for NN training. NNs trained with separate speech and noise losses have been demonstrated to be beneficial for SE. For example, Xu et al. (26) proposed a masking-based SE, in which the NN to estimate the masking gain was trained with a loss function containing separately computed speech distortion and residual noise. Objective evaluations demonstrated that NN trained with the new loss outperformed the one trained with traditional MSE loss, and the best results were attained when the speech and noise losses were equally combined.

As for CI, due to its coarse imitation of the normal auditory system, the recipients obtain an electric hearing much different from the acoustic hearing of NH people. A well-recognized property of electric hearing is that the recipients are more tolerant of speech distortion but very sensitive to noise (10, 27–29). In contrast, NH people are more sensitive to distortion than noise (27, 30). In addition, different CI recipients have noticeable individual differences due to hearing experience, devices, surgery, physiological conditions, etc. Therefore, the individualized perceptual sensitivity to noise and distortion should be considered in designing SE front ends for patients with CI, and a more sophisticated combination of the two losses should be investigated.

This study aims (1) to investigate perceptual sensitivities of the CI recipients to noise and distortion, and will such sensitivities vary across different noise conditions? and (2) to design an effective SE front end with the knowledge of such sensitivities of CI recipients. We developed a DL-based SE as a front end to the signal processing strategy of CIs. A long-short term memory (LSTM) network was trained to estimate the T-F masking gains. Instead of the MSE, a loss function similar to that in Xu et al. (26) was adopted for network training. By adjusting the weights for trading off the speech distortion and the noise residue, their contributions to speech intelligibility for CI recipients were investigated, upon which an LSTM trained with preference-biased-loss was developed. Finally, a set of subjective experiments was conducted to evaluate the system performance.

## Algorithm Description

### SE Based on Time-Frequency Masking

Assuming that speech and noise are additive in the time domain, i.e.,


(1) $$\begin{array}{l} y\left(n\right)=s\left(n\right)+d\left(n\right) \end{array}$$

where *y*(*n*), *s*(*n*), and *d*(*n*) denote noisy speech, clean speech, and noise, respectively. Since speech is a short-term stationary signal, the frequency domain representation of (Equation 1) can be obtained by applying short-time Fourier transform (STFT) to the time signals, i.e.,


(2) $$\begin{array}{l} Y\left(t,\;f\right)=S\left(t,\;f\right)+D\left(t,\;f\right) \end{array}$$

where *t* and *f*denote the index of the time frames and the frequency bins for each T-F unit.

Wiener filtering (WF) has been one of the most widely implemented SE methods to estimate *S*(*t, f*) from *Y*(*t, f*). Given speech and noise uncorrelated, a gain function *G*(*t, f*) to suppress the noise can be written as


(3) $$\begin{array}{l} {G\left(t,\;f\right)=\left(\frac{|S\left(t,\;f\right){|}^{2}}{|S\left(t,\;f\right){|}^{2}+|D\left(t,\;f\right){|}^{2}}\right)}^{1/2}={\left(\frac{|S\left(t,\;f\right){|}^{2}}{|Y\left(t,\;f\right){|}^{2}}\right)}^{1/2} \end{array}$$

Assuming the effect of phase distortion is negligible, the target speech spectra can be estimated by


(4) $$\begin{array}{l} Ŝ\left(t,f\right)=G\left(t,f\right)\cdot Y\left(t,f\right)=G\left(t,f\right)\cdot |Y\left(t,f\right)|\cdot e^{j\varphi_{Y}\left(t,f\right)} \end{array}$$

where φ_*Y*_(*t, f*) is the phase of the noisy speech.

Time-frequency masking, first proposed for speech separation in computational auditory scene analysis tasks, has been demonstrated to be the most successful in SE tasks. WF can be regarded as T-F masking for noise suppression. Essentially, the masking gain as in (Equation 3) provides the optimal filtering in the sense of minimized MSE, given an accurate estimation of noise or SNR. Unfortunately, such an accurate noise/SNR estimation is usually not an easy task.

**Figure 1:**
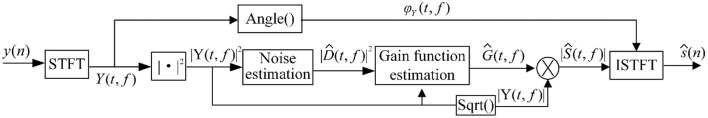
The diagram for WF-based SE.

Figure 1 shows the diagram for a WF-based SE. As illustrated, a noise estimation from the noisy speech spectra is required for computing the masking gain.

### Deep Learning-Based T-F Masking for SE

In the DL-based SE, the masking gain is computed from a pre-trained NN. NNs have been known for their powerful learning ability, given enough training data. Therefore, given a well-trained NN, the gain can be reliably computed from the noisy input without an explicit noise/SNR estimation.

**Figure 2:**
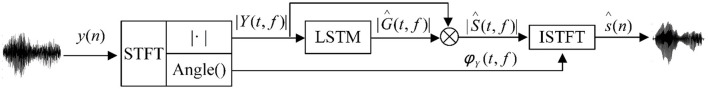
The diagram for SE with DL-estimated T-F masking gain.

Figure 2 shows the diagram for DL-based SE in which the masking gain is computed from the noisy input by a pre-trained LSTM. Here, LSTM is adopted for its superiority in modeling sequential signals like speech over other networks. As shown, the pre-trained LSTM takes the noisy spectral magnitude,|*Y*(*t, f*)|, as input and output of the masking gain, *Ĝ*(*t, f*), which multiplies |*Y*(*t, f*)| to generate a denoised spectral magnitude, |*Ŝ*(*t, f*)|. Finally, the inverse STFT (ISTFT) is employed to recover the time-domain signal from the enhanced magnitude spectra and noisy phase spectra. Unlike the WF, no explicit noise or SNR estimate is required, as the gain is directly estimated by the LSTM.

### Loss Functions for NN Training

Many factors affect the performance of an NN, including the network structure, training strategy, optimization method, etc. This study investigates the effect of different loss functions, i.e., the way measuring the difference between NN output and the target signal, on their performance on NN training.

The most adopted loss is the MSE given by


(5) $$\begin{array}{l} J_{MSE}=\frac{1}{T\cdot F}\underset{t}{\sum }\underset{f}{\sum }{\left(\left|Ŝ\left(t,f\right)|-|S\left(t,f\right)\right|\right)}^{2} \end{array}$$

where *T* and *F* are the total numbers of time frames and frequency bins, respectively.

As known, noise suppression in any SE may induce an inevitable distortion to the target speech. Usually, the more noise is suppressed, the more speech gets distorted. The MSE loss in (Equation 5) computes the overall errors, including both speech distortion and noise residue. It forces the DL-based SE system to output estimated speech that is statistically and objectively *optimal* with respect to the data. However, speech perception is subjective, and the data-level objective optimum might not necessarily result in a perceptual optimum. Specifically, perceptual sensitivity to noise and speech distortion varies across different noise conditions and different subjects. Therefore, the NN may benefit from being trained with a loss function trading off the speech distortion and the noise residue.

We introduce a new loss function combining weighted speech distortion and noise residue, noted as weighting loss (WL), to train the LSTM. The WL is given as (26).


(6) $$\begin{array}{l} J_{WL}=\alpha \frac{1}{T\cdot F}\sum_{t}\sum_{f}{\left(|\tilde{S}(t\;,f)|-|S(t\;,f)|\right)}^{2}\\ \;+(1-\alpha )\frac{1}{T\cdot F}\sum_{t}\sum_{f}{\left(|\tilde{D}(t\;,f)|\right)}^{2} \end{array}$$

where $|\tilde{S}\left(t,f\right)|=Ĝ\left(t,f\right)\cdot |S\left(t,f\right)|\;$is the distorted speech spectrum, i.e., the remaining target speech components after masking, $|\tilde{D}\left(t,f\right)|=Ĝ\left(t,f\right)\cdot |D\left(t,f\right)|\;$is the noise residue, and α is the weighting factor. Given α = 1, the loss *J*_*WL*_forces the SE system to retain the target speech components as much as possible, regardless of whether it suppresses the noise components. On the other hand, if α = 0, the system suppresses the noise as much as possible, regardless of the speech distortion. That is, the remaining noise residue and the induced speech distortion can be traded off by adjusting the parameter α. From (Equation 5) and (Equation 6), it is easy to infer that α = 0.5 does not give *J*_*WL*_ identical to *J*_*MSE*_ in general, and only when the clean speech could be perfectly estimated (i.e., |*Ŝ*(*t, f*)| = |*S*(*t, f*)|), α = 0.5 gives *J*_*WL*_ = *J*_*MSE*_ = 0.

**Figure 3:**
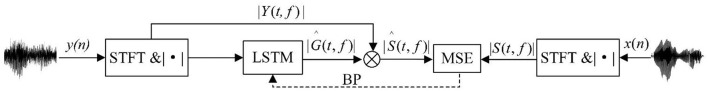
The diagram for training the LSTM with MSE loss.

**Figure 4:**
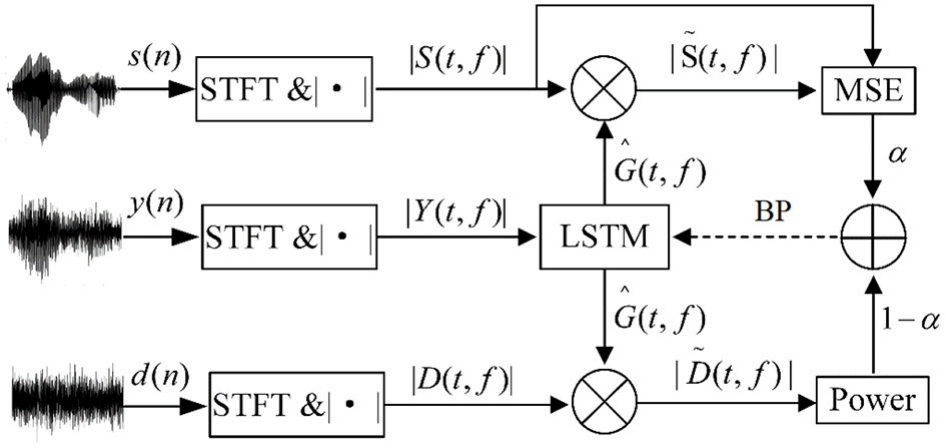
The diagram for the training of the LSTM with weighting loss.

Figures 3, 4 give the diagrams for the training of the LSTM with the respective MSE- and WL-based loss functions. As shown, the LSTMs are optimized iteratively by the backpropagation (BP) algorithm with the respective losses.

## Experimental Setting

### Speech Materials

Two speech corpora, i.e., an open-access Chinese speech database built by Tsinghua University, THCHS-30 (31) and the Mandarin hearing in noise test, MHINT-M (32), were adopted for the experiments. THCHS-30 is a Mandarin speech database widely used to develop DL-based speech systems. It contains three subsets, i.e., training, development, and test sets, consisting of 10,000, 893, and 2,495 utterances, respectively. MHINT-M is a Mandarin speech database designed for the subjective listening test. It contains 14 lists, 12 for formal tests and two for practice. There are 20 utterances in each list and 10 Mandarin syllables in each utterance. The noisy speeches were generated by additively mixing the clean ones with two noise samples, SSN and Babble. The SSN noise was generated by shaping (multiplying) a white noise spectrum with an averaged speech envelope. The Babble noise was taken from the NOISE-92 database (33). The duration of SSN and Babble noises are about 10 and 4 min, respectively. In this study, the training and validation of NNs in the training stage used the training and the development sets of THCHS-30, and all tests used the speech signals in MHINT-M. All speech and noise signals were downsampled to 16 kHz for the experiments.

There were, in total, 10 noisy conditions for the training, i.e., two noises (SSN and Babble), each at five SNRs (from 0 to 20 dB in a step of 5 dB), in generating the noisy speech. Each utterance in the training and development sets of THCHS-30 was randomly mixed with a noise segment (randomly picked out from the whole noise recording) in one of the 10 conditions such that there are 10,000 and 839 noisy utterances used for NN training and validation, respectively.

There were 62 noisy conditions for the test, i.e., two noises, each at 31 SNRs (from −10 to 20 dB in a step of 1 dB), for noisy speech generation. Each utterance in MHINT-M was mixed with a noise segment in all 62 conditions. Note that the whole set of noisy speech was used for subjective evaluations, but only a subset, with SNRs from −5 to 15 dB in a step of 5 dB, was selected for objective evaluations.

### SE Systems to be Evaluated

Several DL-based SE systems were developed to examine how the cost function could affect the DL-based SE for CI. Two loss functions introduced in section Loss Functions For NN Training, i.e., the MSE loss and the weighting loss (with different weights), were used for network training. In addition, Wiener filtering-based SE was also developed for comparison, from which the performance gap between the traditional and the DL-based SEs can be shown.

**Wiener filtering (WF)**: instead of the traditional WF, a parametric WF (34) was adopted as the SE front end to CI. The gain function is given as


(7) $$\begin{array}{l} G\left(t,f\right)=\max\left(\frac{|Y\left(t,\;f\right){|}^{2}-\alpha \left(t\right)|\hat{D}\left(t,\;f\right){|}^{2}}{|Y\left(t,\;f\right){|}^{2}},0.01\right) \end{array}$$

where $|\hat{D}\left(t,\;f\right){|}^{2}$is estimated by an energy-based voice activity detector, the floor parameter 0.01 is set to avoid negative or very small gain, α(*t*) is a factor to avoid the overestimation of noise and is computed based on the local *a posterior* SNR [(*SNR*_*post*_(*t*))], i.e.,


(8) $$\alpha (t)=\{\begin{array}{c} 3.125,\;SNR_{post}(t)<0dB\\ \frac{-1.875}{20}SNR_{post}(t)+3.125,\;others\;\\ 1.25,\;SNR_{post}(t)>20dB \end{array}$$

where $SNR_{post}\left(t\right)=10\log_{10}\frac{\underset{f}{\sum }|Y\left(t,\;f\right){|}^{2}}{\underset{f}{\sum }|\hat{D}\left(t,\;f\right){|}^{2}}$. In this experiment, WF was implemented with the source code of the parametric WF download from https://github.com/dpwe/pitchfilter. More details of the baseline can be referred to the webpage.

**T-F masking with gains computed by MSE-trained LSTM (MSE-MASK)** The LSTM consisted of three layers, i.e., an input layer with 256 LSTM units, a hidden layer with two fully connected (FC) layers (512 neural units per layer), and an output layer with 256 FC units such that the output has the same dimension as the input. LeakyReLU activation function was applied to the input and hidden layers. Sigmoid activation function was applied to the output layer. The parameters of networks were optimized by Adam optimizer with an initial learning rate of 0.005. When the loss did not decline for two consecutive epochs, the learning rate was reduced to half until < 0.00001. The model was trained for 60 epochs. The validation was implemented after each training epoch. Finally, the best model, i.e., the one with the minimum loss among all the validated ones, was selected for tests.

The long-short term memory was trained with all noisy speech covering all the 10 noise conditions mentioned in section Speech Materials. To train the LSTM, T-F spectra of noisy speech, |*Y*(*t, f*)|, and their corresponding clean spectra, |*S*(*t, f*)|, were served as the input features and the training labels, respectively. To generate the feature, each speech signal was first segmented into short frames by a Hanning window with a 32-ms length and 16-ms shift. Then, a 512-point fast Fourier transform was applied to each frame, and a 256-dimensional feature was constructed with the magnitude spectra of nonnegative frequency components.

**T-F masking with gains computed by WL-trained LSTM (WL-MASK):** The training and validation processes for LSTM were the same as in MSE-MASK, except that the weighting loss *J*_*WL*_, instead of the MSE loss, was used. To investigate the effect of the weighting parameter α, we repeated the training process nine times, each with a specific α from 0.1 to 0.9 in a step of 0.1. That is, there were, in total, nine NNs trained with different α. For each α, an LSTM was trained with all noisy speech covering all the 10 noise conditions mentioned in section Speech Materials.

**Figure 5:**
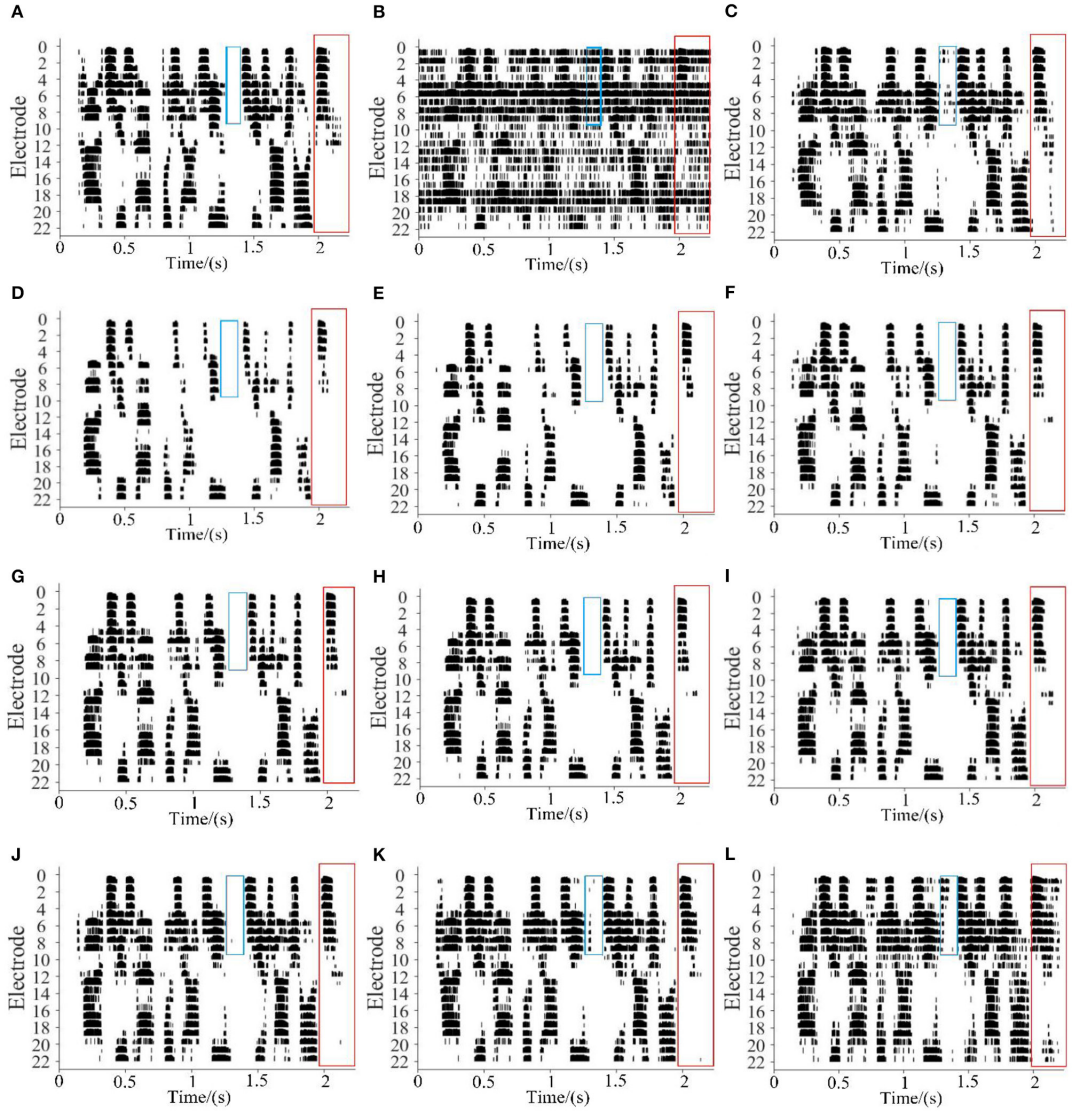
The electrodogram examples of different systems, **(A)** clean speech, **(B)** noisy speech, **(C)** MSE-MASK enhanced, and **(D–L)** WL-MASK with α across from 0.1 to 0.9.

Figure 5 shows the electrodograms extracted from speech signals generated from the same speech utterance with different noisy processing. The electrodograms were generated by processing the acoustical signal with the CCi-Mobile, a CI research development platform developed by CI-Lab at the University of Texas at Dallas (35). Figures 5A,B are for clean speech, and that corrupt by SSN at 0 dB SNR (Figure 5C) is for the denoised speech with MSE-MASK DL SE, and (Figures 5D–L) are for the denoised speeches with WL-MASK DL SEs with α = 0.1, 0.2, ⋯, 0.9, respectively. Two spectro-temporal regions in the electrodograms are marked with red boxes and blue boxes for better illustration. As shown, the noise seriously corrupts the electrodogram. All the SE processings suppress the noise to a certain degree and, at the same time, introduce some speech distortion. The MSE-MASK seems to have a balanced speech distortion and noise residue. As for WL-MASK, noise is mostly suppressed, and a large number of speech components are deleted at small α; as α increase, speech components are mostly retained, so as the noise components. Therefore, user-preference-dependent noise-distortion tradeoff could be achieved by properly selected α.

To further investigate the effect of α in trading off the speech distortion and residual noise in the electrodograms, we computed and compared the current units of the enhanced electrograms and the clean ones. The CI speech processor maps the subband envelopes into currents from 0 unit to 255 units. We consider distortion happens when the current unit of the enhanced electrodogram is lower than that of the clean one; otherwise, there exists residual noise. The degree of speech distortion and noise residue is computed as,


(9) $$\begin{array}{l} C_{dis}=\frac{1}{I\cdot T}\underset{i}{\sum }\underset{t}{\sum }\max\left\{0,C_{ref}\;\left(i,t\right)-C\left(i,t\right)\right\} \end{array}$$

(10) $$\begin{array}{l} C_{res}=\frac{1}{I\cdot T}\underset{i}{\sum }\underset{t}{\sum }\max\left\{0,C\left(i,t\right)-C_{ref}\;\left(i,t\right)\right\} \end{array}$$

where *i* and *t* represent the indices for electrode channels and time frames, *I* and *T* are the numbers of electrode channels and time frames, *and C*_*ref*_(*i, t*) and *C*(*i, t*) are the current units of the clean electrodograms and the enhanced ones.

**Figure 6:**
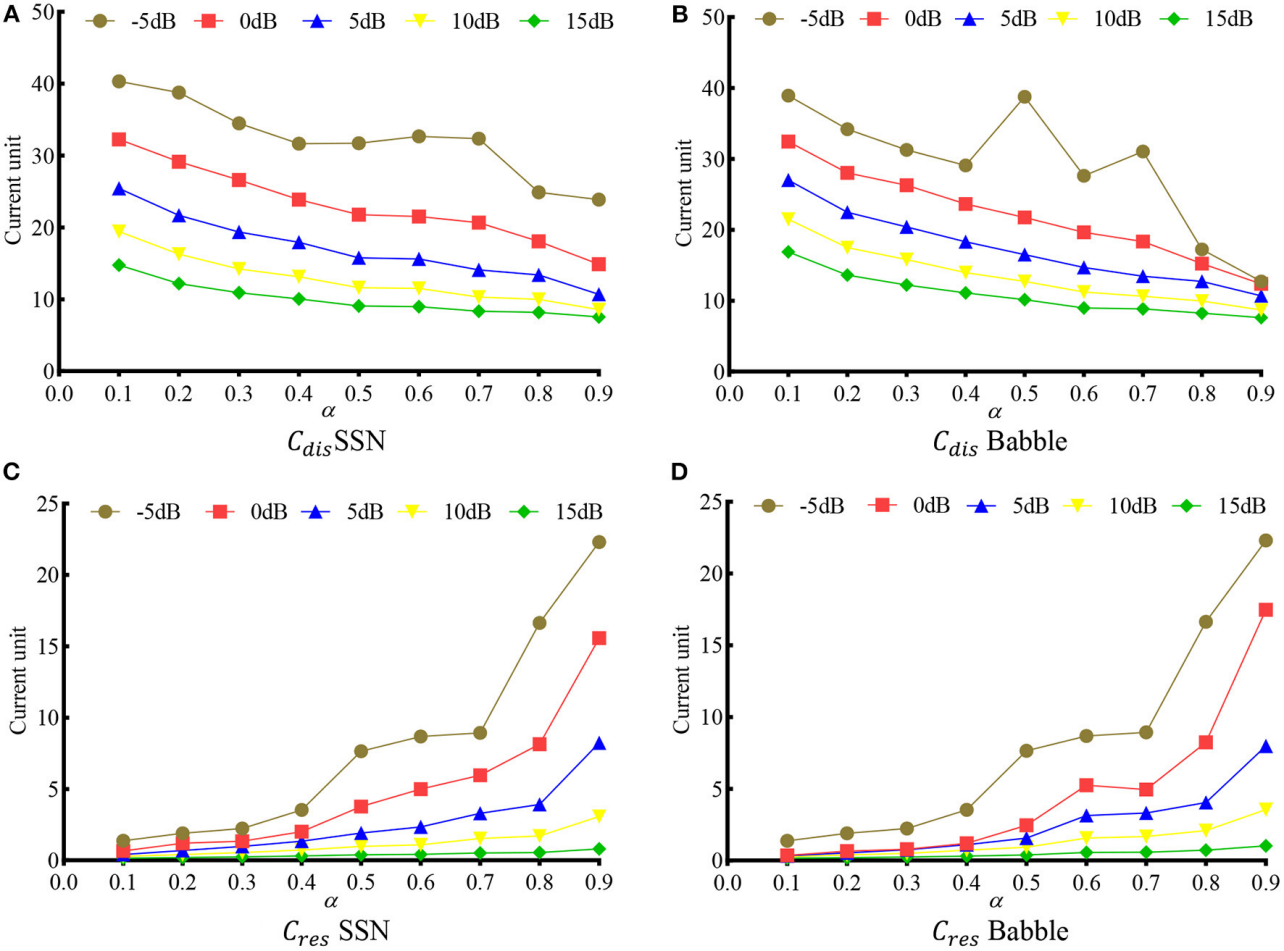
Speech distortion (*C*_*dis*_) and noise residue (*C*_*res*_) as a function of α in various noise conditions. **(A)** *C*_*dis*_ in SSN, **(B)** *C*_*dis*_ in Babble, **(C)** *C*_*res*_ in SSN, and **(D)** *C*_*res*_ in Babble.

Figure 6 shows the *C*_*dis*_ and *C*_*res*_ at different α. Ten noise conditions, i.e., two noise types (SSN and Babble), each at five SNRs (−5, 0, 5, 10, and 15 dB), were evaluated. It is clear that, as α increases, the distortion decreases monotonically, and the noise residue increases monotonically in most noise conditions. The only exceptions happen at *C*_*dis*_ in −5 dB SSN, *C*_*dis*_ in −5 dB Babble, and *C*_*res*_ in 0 dB SSN, where some fluctuations appear at around α = 0.6. The fluctuation in −5 dB might be because the network has not seen a −5 db SNR during the training.

## Objective Evaluation

### Methods

The envelope-based correlation measure (36), an objective metric to evaluate speech intelligibility by CI recipients, was adopted to measure the performance of different SE systems. In this study, the CCi-Mobile platform was adopted for extracting channel envelopes with the Advanced Combination Encoder (ACE) strategy (37). Given two versions of a speech, e.g., a target one and its distorted one, ECM computes the correlation of their extracted channel envelopes, which will modulate the pulsatile carriers and stimulate the electrodes. In the signal processing strategy of CI, the recipient-dependent MAP parameters are used in computing the channel envelopes. Therefore, ECM computed from such subject-dependent envelopes well represents the speech intelligibility of the corresponding CI recipient (36). The score of ECM is between 0 and 1. The higher ECM, the better intelligibility.

In this experiment, noisy test speech signals were first denoised by different SE front ends and then processed by the ACE strategy in the CCi-Mobile CI research development platform. In addition, the sample MAP file provided in the CCi-Mobile demo system was used in generating the envelopes. ECM was computed on each pair of extracted envelopes, i.e., reference one and distorted one.

### Results

Table 1 shows the mean ECM scores for SSN-corrupted noisy speech and their denoised versions with different SE front ends, i.e., WF, MSE-MASK, and WL-MASK with α = 0.1, 0.2, ⋯, 0.9. For WL-MASK, the highest score among the nine α is highlighted in red fonts. As illustrated in Table 1, all SE front ends achieved a certain ECM gain over noisy speech, except for those highlighted in blue fonts, i.e., WF at high SNRs (10 and 15 dB) and WL-MASK with α = 0.1 at SNR of 15 dB. Both DL-based SEs outperformed WF in all SNRs. The performance of the WL-MASK front end varied at different α. Nevertheless, there always exists some α, although the values vary at different SNRs, with which the WL-MASK front end achieved better performance than MSE-MASK.

**Table 1:** The mean ECM score results for different systems under SSN.

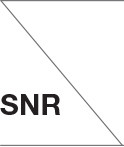	**Noisy**	**WF**	**MSE-MASK**	**WL-MASK**								
				**α = 0.1**	**α = 0.2**	**α = 0.3**	**α = 0.4**	**α = 0.5**	**α = 0.6**	**α = 0.7**	**α = 0.8**	**α = 0.9**
−5 dB	0.143	0.190	0.235	**0.313**	0.256	0.254	0.264	0.282	0.243	0.258	0.234	0.226
0 dB	0.238	0.296	0.456	0.430	0.451	0.485	**0.493**	0.485	0.492	0.489	0.458	0.365
5 dB	0.365	0.428	0.596	0.502	0.556	0.584	0.599	0.586	**0.607**	0.604	0.588	0.503
10 dB	0.512	**0.507**	0.702	0.585	0.652	0.676	0.691	0.683	**0.707**	0.702	0.697	0.643
15 dB	0.712	**0.560**	0.801	**0.673**	0.738	0.764	0.781	0.777	0.800	0.800	**0.805**	0.782

*The red bold indicates the best score under each SNRs. The blue bold indicates that worse than noisy condition*.

Table 2 shows the mean ECM scores for Babble-corrupted noisy speech and their denoised versions with different SE front ends. Most SE front ends achieved a certain ECM gain over noisy speech, except for those highlighted in blue fonts. Both DL-based SEs outperformed WF in all SNRs. The performance of the WL-MASK front end varied at different α. Unlike in SSN, in SNR of 5 and 10 dB, the WL-MASK with optimal α showed comparable performance to MSE-MASK.

**Table 2:** The mean ECM score results for different systems under Babble.

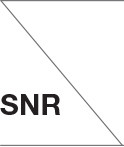	**Noisy**	**WF**	**MSE-MASK**	**WL-MASK**								
				****α** = 0.1**	****α** = 0.2**	****α** = 0.3**	****α** = 0.4**	****α** = 0.5**	****α** = 0.6**	****α** = 0.7**	****α** = 0.8**	****α** = 0.9**
−5 dB	0.201	0.228	0.291	0.276	0.293	**0.315**	0.310	0.313	0.292	0.283	0.274	0.253
0 dB	0.295	0.322	0.447	0.413	0.437	**0.464**	0.462	0.463	0.450	0.438	0.424	0.374
5 dB	0.427	0.440	0.592	0.508	0.557	0.583	**0.590**	**0.590**	0.589	0.586	0.576	0.518
10 dB	0.602	**0.528**	0.719	**0.600**	0.664	0.691	0.708	0.704	0.717	**0.719**	0.716	0.676
15 dB	0.789	**0.577**	0.825	**0.695**	**0.760**	**0.787**	0.810	0.801	0.824	0.829	**0.833**	0.809

*The red bold indicates the best score under each SNRs. The blue bold indicates that worse than noisy condition*.

Tables 1, 2 tell that, although the optimal α varies across SNRs, it generally increases as the SNR increases. Note that α is the weight for loss induced by speech distortion. Therefore, a larger α forces the network to output less distorted speech; in contrast, a smaller α forces the network to suppress more noise. At low SNRs, noise is the dominant component in noisy speech. Hence, the network must put more attention on noise suppression to improve speech intelligibility. On the other hand, speech dominates the noisy signal at high SNRs, and a larger α is preferred to avoid significant speech distortion. Note that, even for SNR of 0 dB, where speech and noise have the same energy, ECM evaluation shows that noise suppression biased α (0.4 for SSN, 0.3 for Babble) achieved better results. It is reasonable since it is well-known that, unlike NH people, CI recipients are much more sensitive to noise than distortion.

## Subjective Evaluation: Vocoder Simulation With NH Subjects

### Methods

Speech reception threshold in noise (38), an SNR level at which the listener could correctly recognize 50% of words in a sentence, was adopted to investigate how the speech distortion and noise residue trading-off would affect the intelligibility of the enhanced speech.

Ten college students, all are normal hearing (pure-tone thresholds not >25 dB HL) and native Mandarin speakers, were recruited with a reward for the test. Each subject underwent 12 SRT measure blocks, each for one of the 12 SE front ends, i.e., Noisy (no SE), WF, MSE-MASK, and WL-MASK with nine α. The 12 test lists from MHINT-M were used, each for a block. Before the formal test, the subject had taken a practice session with the two practice lists in MHINT-M. Due to the limit of speech materials, each subject was tested with one noise type, either SSN or Babble.

The noisy speech signals were first processed by the SE front ends. Then, the vocoded speeches were generated from a Gaussian-enveloped-tone vocoder (39), which directly mapped the electric stimulus of a CI to the acoustic signal. Meng et al. (39) and Kong et al. (40) have demonstrated that this vocoder better predicts the potential CI performance than classical continuous-carrier vocoders. The CCi-Mobile platform was used to generate electric stimuli (electrodogram) of the ACE strategy, where the n-of-m strategy was set to 8 of 22, which is the same as that of Meng et al. (39). The vocoded speech was presented diotically to the NH subjects *via* a Sennheiser HD650 headphone in a soundproof booth.

In each block, SRT was measured with an adaptive procedure using a 20-utterances test list. SNR was adaptively modified in a one-down, one-up way (41). An utterance was “intelligible” when more than half of its syllables were repeated correctly by the subject. The SNR was initialized at 12 dB and changed by 4 dB before the second reversal and by 2 dB afterward. Each sentence could be replayed up to three times upon the request of the subjects. The SRT was computed as the average of the intermediate SNRs of the last six reversals to reduce the measurement deviation.

### Results

**Figure 7:**
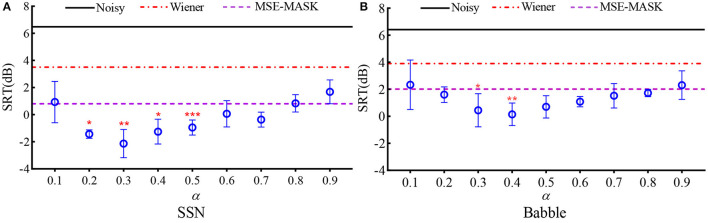
SRTs for different SE front ends with the vocoded speech, **(A)** SSN and **(B)** Babble. The circles represent the means, and the error bars represent the standard deviations for each WL-MASK. The asterisk represents the result of pairwise comparison of MSE-MASK and WL-MASK, * indicates *p* < 0.0332, ** indicates *p* < 0.0021, and *** indicates *p* < 0.0001.

Figure 7 shows the SRTs for the 12 SE front ends, the left panel for SSN, and the right panel for Babble. For WL-MASK, the mean and standard deviation of SRT for each α are depicted, while for Noisy, WF, and MSE-MASK, mean SRTs are given as constant lines for comparison. As shown, the DL-based SEs outperform the unprocessed noisy speech and that with WF in both noises. Furthermore, by properly trading off the errors induced by speech distortion and residual noise, WL-MASK SE attained lower SRTs than MSE-MASK. In SNN, the best SRT of −2 dB was obtained at α = 0.3 and, in Babble, SRT of 0 dB was obtained at α = 0.4. The results coincided with the ECM ones in Tables 1, 2, where the best ECMs were obtained at α = 0.4 (SSN) and 0.3 (Babble) at SNR of 0 dB. One-way repeated measure analysis of variance (RM-ANOVA) was applied to analyze the influences of different factors. The results show that there is a significant difference among different DL-SE methods {[F_(9, 36)_ = 1.262, *p* < 0.001]}. Dunnett's test was used to pairwise compare MSE-MASK and different WL-MASKs. In SSN, WL-MASK with α from 0.2 to 0.5 obtained significant improvement over MSE-MASK (*p* < 0.0332 at α = 0.2 and 0.4; *p* < 0.0021 at α = 0.3; *p* < 0.0001 at α = 0.5). In Babble, WL-MASK with α = 0.3 and 0.4 significantly outperformed MSE-MASK (*p* < 0.0332 at α = 0.3; *p* < 0.0021 at α = 0.4).

## Subjective Evaluation With CI Recipients

### Participants

Nineteen CI recipients were recruited for the evaluation. They were all single-side implanted with the CI24M series of Cochlear corporation and could normally communicate in Mandarin in quiet environments. Table 3 lists the individual information of the subjects. Subjects with processors having noise reduction built-in, i.e., Nucleus 6, Nucleus 7, and Kanso, turned off the noise reduction function during the evaluation. Before the evaluation, each subject read the informed consent and agreed with it. All the subjects were paid after the listening test. This subjective evaluation has been approved by the Medical Ethics Committee of Shenzhen University.

**Table 3:** The individual subject information for speech recognition evaluation.

**Participant**	**Age at testing (years old)**	**Etiology**	**CI experience (years)**	**Implanted side**	**Processor**
C1	23	Drug induced	20	R	Freedom
C2	23	Drug induced	17	R	Freedom
C3	23	Drug induced	17	R	Sprint
C4	22	Acute meningitis	3	L	Freedom
C5	37	Otitis media	10	R	Esprit 3G
C6	21	Otitis media	12	R	Nucleus 7
C7	26	Drug induced	20	R	Nucleus 5
C8	17	Unknown	14	R	Nucleus 5
C9	12	Unknown	10	R	Kanso
C10	13	LAVS	7	R	Nucleus 5
C11	16	Unknown	12	L	Nucleus 6
C12	15	Drug induced	14	R	Esprit 3G
C13	11	Unknown	8	R	Freedom
C14	28	Drug induced	18	L	Kanso
C15	17	Unknown	16	L	Nucleus 6
C16	12	LAVS	7	R	Nucleus 6
C17	12	Unknown	4	R	Nucleus 5
C18	16	Drug induced	14	R	Nucleus 7
C19	17	Unknown	12	R	Nucleus 5

*CI, cochlear implant; F, female; L, left; M; male, and R, right*.

### SRT Test

Seven CI recipients, i.e., C1–C7 as listed in Table 3, participated in the SRT test. Each subject underwent 10 blocks, each measuring the SRT with one of the 10 experimental conditions: five front ends (Noisy, WF, MSE-MASK, and WL-MASK with α of 0.3 and 0.4), each for two noises (SSN and Babble). Here, only two α*s* for WL-MASK were tested because those, as illustrated by Figure 3, SRTs were mostly around 0 dB, where the optimal α was 0.3 or 0.4. Each block used a 20-utterances list in MHINT-M as the original speech materials. The processed signals were presented to subjects *via* two Genelec 8030A loudspeakers at a comfortable level (about 65 dB SPL) in a soundproof booth. The two loudspeakers were placed front-right or front-left to the participant such that the participants with either left or right side implanted could be equally presented. Before the formal test, each subject had finished two practice blocks. The SRT search process was the same as the vocoder simulation experiments.

**Figure 8:**
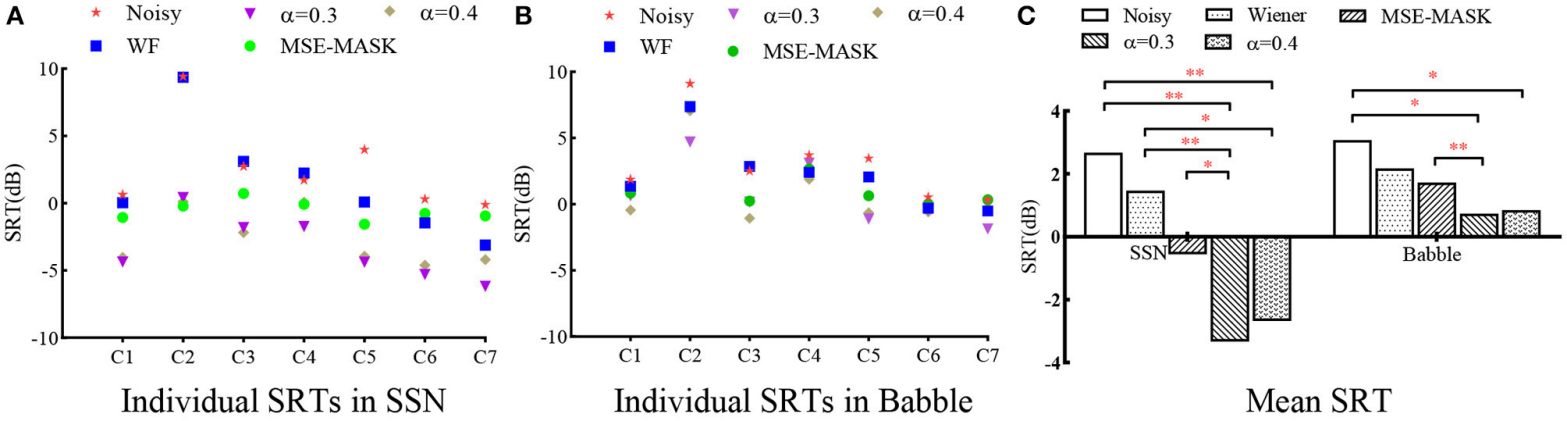
CI users' SRT results for different systems. **(A)** Individual results in SSN, **(B)** individual results in Babble, and **(C)** statistical analysis of the different methods. The asterisk in **(C)** represent significance analysis results. ^*^ indicates *p* < 0.0332, ^**^ indicates *p* < 0.0021, ^***^ indicates *p* < 0.0001.

Figure 8 shows the SRTs for different SE front ends measured on the seven CI subjects. The left and middle panels give the individual results in SSN and Babble, respectively, and the right panel gives the statistical results on all the subjects. For the individual results, most subjects achieved better SRTs with enhanced speech than noisy speech, except that WF and Noisy performed comparably for C2, C3, and C4. WL-MASK with α= 0.3 and 0.4 performed better than MSE-MASK for most subjects except C2. Two-way repeated measure ANOVA (RM-ANOVA) was applied to analyze the influences of different factors. Results showed that there were significant interactions between noise types and SE methods [F_(1.56, 9.361)_ = 7.152, *p* = 0.02], and there were significant differences within SE methods [F_(1.825, 10.97)_ = 19.69, *p* < 0.001] and within noise types [F_(1, 6)_ = 43.6, *p* < 0.001]. Tukey's multiple comparisons test was used for pairwise comparison. In SSN, WL-MASK with α = 0.3 and 0.4 significantly improved speech intelligibility against Noisy (both *p* < 0.0021), and significantly outperformed WF (at least *p* < 0.0332), and the superiority of WL-MASK over MSE-MASK was significant (*p* < 0.0332) with α= 0.3 but not significant with α= 0.4. In Babble, WL-MASK with α= 0.3 showed significant superiority over Noisy (*p* < 0.0332) and MSE-MASK (*p* < 0.0021), WL-MASK with α= 0.4 showed significant superiority over Noisy (*p* < 0.0332), but its superiority over WF and MSE-MASK was not significant.

### Speech Recognition (SR) Test

The ECM results show that the optimal α increases as the SNR increases. To verify this phenomenon, we conducted a speech recognition test to investigate how the trading-off weight α can maximize the SE gain for CI recipients in different noise conditions.

Twelve CI recipients, i.e., C8–C19 as listed in Table 3, participated in the SR test. Due to the limitation on speech materials, only three different α*s*, i.e., 0.3, 0.5, and 0.7, were tested. Each subject conducted 12 SR blocks, each with one of the 12 test conditions: three SNRs (0 dB, 5 dB, and 10 dB) ^*^ four systems (Noisy, WL-MASK with the three α*s*)^*^ one noise type (either SSN or Babble). Each block randomly took one of the 12 lists in the MHINT-M database as the speech materials. The utterance order was also random in each list. The processed speeches were presented to the subjects in the same way as in the SRT test. Before the formal measurement, each subject had finished two practice blocks in 10 dB noisy condition. In each block, the sentences were presented in random order. Each sentence can be replayed up to three times upon the request of the subjects. The mean word recognition rate (WRR) of the 20 utterances was calculated as the final result in each trial.

**Figure 9:**
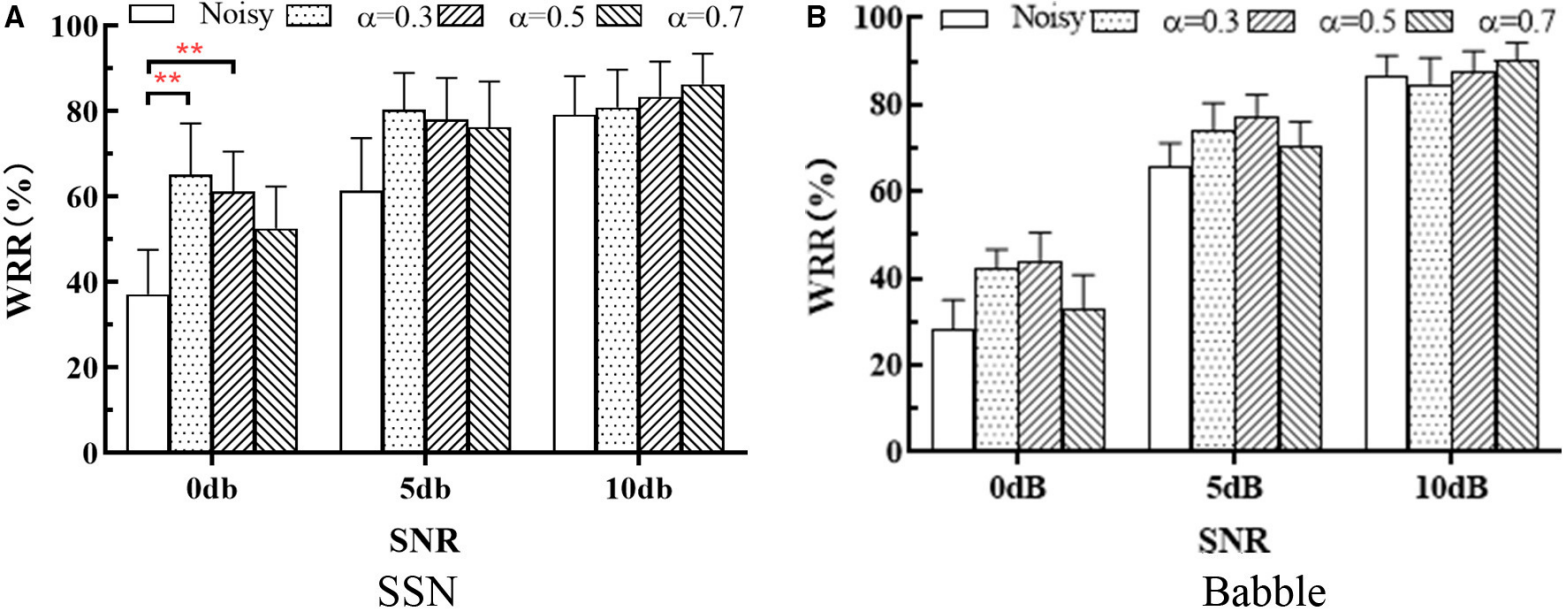
WRR of different systems at different SNRs in **(A)** SSN and **(B)** Babble. The bars show the mean WRR of all the subjects, and error bars indicate the standard deviations. The asterisk indicates a significant level, * indicates *p* < 0.0332, ** indicates *p* < 0.0021, and *** indicates *p* < 0.0001.

Figure 9 shows the mean and standard deviation of WRR (over all the subjects) at different SNRs. The same methods as in section SRT Test were used for significance analysis. RM-ANOVA test showed that, in SSN, there was no significant [F_(1.267, 6.333)_ = 5.375, *p* = 0.05] interaction between SNRs and SE methods, there were significant differences within SE methods [F_(2.026, 10.13)_ = 19.65, *p* < 0.001] and within SNRs [F_(1.404, 7.003)_ = 61.46, *p* < 0.001]; in Babble, there was no significant [F_(1.941, 9.704)_ = 3.421, *p* = 0.08] interaction between SNRs and SE methods, and there were significant differences within SE methods [F_(1.613, 8.064)_ = 11.97, *p* = 0.005] and within SNRs [F_(1.616, 5.803)_ = 80.38, *p* < 0.001]. Tukey's multiple comparisons tests showed that WL-MASK has no significant performance improvement over Noisy in all noise conditions except for 0 dB SSN, where WL-MASK with α = 0.3 and 0.5 achieved significant improvement (*p* = 0.007 and 0.004).

## Discussions

The residual noise and speech distortion in the enhanced signal generally determine the effect of SE. It is well-known that, unlike NH people, CI recipients are more sensitive to noise than to speech distortion in their daily speech perception. The distinct noise- and distortion-perception properties of CI have been investigated and adopted to design the enhancement algorithms (10, 29, 42).

In this study, we developed a deep learning-based SE to systematically investigate how the noise residue and speech distortion could affect the intelligibility of the enhanced speech for patients with CI, and how such noise and distortion sensitivities could be adopted for SE system design. An LSTM-based time-frequency masking system was developed as an SE front end to CI, different loss functions were used to train the system such that different levels of residual noise and speech distortion in the output speech signal could be retained for the investigation. Several objective and subjective experiments were conducted to evaluate the performance of the SE system at different loss conditions.

The objective evaluation with the ECM metric (Tables 1, 2) showed that the MSE loss aiming at minimizing the overall difference between the target and enhanced speech signals usually had suboptimal results in both SSN and Babble noises. By training the LSTM with weighting loss, the SE performance varied at different weighting parameters α. In general, smaller α, which tends to remove more noise components (and induce more speech distortion), was preferred for noisy speech with lower SNRs in both SSN and Babble. Whatever the SNR, there exist some specific α with which the WL-MASK system outperformed the MSE-MASK system. The superiority was more evident in SNN than in Babble. In particular, for SNR of 0 dB, where speech and noise had the same energy, α of 0.4 (in SSN) and 0.3 (in Babble) achieved better results.

Vocoder simulation evaluation with SRT in noise by NH people (Figure 7) gave consistent results to that by ECM. Compared with MSE-MASK, WL-MASK had an SRT benefit of about 2.8 dB in SSN (with α = 0.3) and about 1.7 dB in Babble (with α = 0.4). Compared to Noisy, WL-MASK had an SRT benefit of about 8.6 dB in SSN and about 6.3 dB in Babble.

SRT in noise test with seven CI users showed similar results. Compared with MSE-MASK, WL-MASK had an SRT benefit of about 3.3 dB in SSN and about 1.2 dB in Babble. Compared with Noisy, WL-MASK had an SRT benefit of about 6 dB in SSN and about 2.2 dB in Babble. The SRT gains obtained by WL-MASK are compatible in both NH and CI tests, except for the case with WL-MASK over Noisy in Babble, where the SRT gains drop from 6.3 dB by NHs to 2.2 dB by CIs. In this study, we did find that Babble noise is a relatively more challenging condition for the NN-based SE systems.

Speech recognition tests with 12 CI recipients showed that the proposed WL-MASK had no significant improvement over Noisy in all noise conditions, except for the low SNR (0 dB) SSN case, although the mean word recognition rates did demonstrate that the preferred α was SNR dependent. The lack of significance in high SNRs might be due to the ceiling effect.

The same noise recording used for training and testing could be a limitation of this work in considering the generation of the NN-based SE for real-world applications. Nevertheless, 4- or 10-min recordings are relatively long enough to cover possible variations of a specific noise type. Therefore, this study could reflect the NN-based SEs performance for CI where NNs are well trained with enough noise data, i.e., all noise had been seen by the NNs after training. It is true that real-world settings could be more challenging, which requires more sophisticated NNs and a much larger amount of training noise as well.

Compared to traditional SE methods, the computation load of a DL-based system should be considered in real-world implementations. Furthermore, the WRR test implies that noise conditions-dependent SE systems need to be pre-trained, and real-time noise estimation is required to maximize the benefit from the noise-dependent SE. These drawbacks might restrict the implementation of the proposed WL-MASK SE in clinical CI systems. Nevertheless, this research indicates that it would be promising to further explore the hearing properties of patients with CI and utilize such properties for designing new signal-processing strategies.

## Data Availability Statement

The original contributions presented in the study are included in the article/supplementary material, further inquiries can be directed to the corresponding author.

## Ethics Statement

The studies involving human participants were reviewed and approved by the Medical Ethics Committee of Shenzhen University. Written informed consent to participate in this study was provided by the participants' legal guardian/next of kin.

## Author Contributions

YK, NZ, and QM contributed to the design and writing of this paper. YK and NZ planned the experimental program, collected data, and wrote the manuscript. All authors agree to submit this version of the article.

## Funding

This work was jointly supported by Guangdong Key Area R&D Project (No. 2018B030338001), National Natural Science Foundation of China (61771320), Guangdong Basic and Applied Basic Research Foundation (2020A1514325010386), and Science and Technology Program of Guangzhou (202102020944).

## Conflict of Interest

The authors declare that the research was conducted in the absence of any commercial or financial relationships that could be construed as a potential conflict of interest.

## Publisher's Note

All claims expressed in this article are solely those of the authors and do not necessarily represent those of their affiliated organizations, or those of the publisher, the editors and the reviewers. Any product that may be evaluated in this article, or claim that may be made by its manufacturer, is not guaranteed or endorsed by the publisher.
